# A Genomic Safe Haven for Mutant Complementation in *Cryptococcus neoformans*

**DOI:** 10.1371/journal.pone.0122916

**Published:** 2015-04-09

**Authors:** Samantha D. M. Arras, Jessica L. Chitty, Kirsten L. Blake, Benjamin L. Schulz, James A. Fraser

**Affiliations:** Australian Infectious Diseases Research Centre and School of Chemistry & Molecular Biosciences, The University of Queensland, Brisbane, Queensland, Australia; University of Minnesota, UNITED STATES

## Abstract

Just as Koch’s postulates formed the foundation of early infectious disease study, Stanley Falkow’s molecular Koch’s postulates define best practice in determining whether a specific gene contributes to virulence of a pathogen. Fundamentally, these molecular postulates state that if a gene is involved in virulence, its removal will compromise virulence. Likewise, its reintroduction should restore virulence to the mutant. These approaches are widely employed in *Cryptococcus neoformans*, where gene deletion via biolistic transformation is a well-established technique. However, the complementation of these mutants is less straightforward. Currently, one of three approaches will be taken: the gene is reintroduced at the original locus, the gene is reintroduced into a random site in the genome, or the mutant is not complemented at all. Depending on which approach is utilized, the mutant may be complemented but other genes are potentially disrupted in the process. To counter the drawbacks of the current approaches to complementation we have created a new tool to assist in this key step in the study of a gene’s role in virulence. We have identified and characterized a small gene-free region in the *C*. *neoformans* genome dubbed the “safe haven”, and constructed a plasmid vector that targets DNA constructs to this preselected site. The plasmid vector integrates with high frequency, effectively complementing a mutant strain without disrupting adjacent genes. qRT-PCR of the flanking genes on either side of the safe haven site following integration of the targeting vector revealed no changes in their expression, and no secondary phenotypes were observed in a range of phenotypic assays including an intranasal murine infection model. Combined, these data confirm that we have successfully created a much-needed molecular resource for the *Cryptococcus* community, enabling the reliable fulfillment of the molecular Koch’s postulates.

## Introduction

In 1884, Robert Koch proposed a set of four criteria designed to establish a causative relationship between a microbe and a disease that defined infectious disease research in the 19^th^ and 20^th^ centuries [1]. Koch’s postulates stated that the causative agent of a disease must be found in abundance in all organisms suffering from the condition, but should not be found in association with healthy individuals. Furthermore, the etiological agents of the disease must be able to be isolated from an infected individual, and the isolated microbe should subsequently cause disease when introduced into a healthy individual [1]. Finally, the etiological agent must be able to be reisolated from the inoculated host, and be identified as identical to the original causative agent.

When the era of molecular genetics began nearly a century later, the need arose to redefine Koch’s postulates. As molecular studies of disease-causing microbes advanced, Stanley Falkow modified Koch’s postulates in order to apply them to the molecular genetics of pathogenicity [2]. Following equivalent criteria to his predecessor, Falkow extended the postulates to require a virulence phenotype under investigation to be associated with a pathogenic species, and the deletion of the genes required for this phenotype to result in a reduction or loss of virulence. Additionally, and perhaps most importantly, the subsequent reintroduction of the wild-type gene should restore pathogenicity, proving that the reduction of virulence observed in the mutant is indeed due to the loss of the gene of interest [2]. These molecular Koch’s postulates now serve as the foundation of molecular genetic studies in pathogenic species.

The experimental methodology employed in creating targeted gene deletion strains differs between species. Common methods include biolistic, chemical and protoplast-mediated transformation, all of which rely on homologous recombination to replace the gene of interest with a selectable marker. The degree of success via these approaches is species-dependent. In *Saccharomyces cerevisiae*, gene deletion through homologous recombination occurs readily, with a 30–60% chance of the desired mutant being created in a single transformation attempt and the entire process taking less than a week [3,4]. In contrast, in species such as *Mycobacterium tuberculosis*, gene deletion mutants are still extremely difficult to create [5].

Importantly, successful molecular genetic analysis of the role of a gene in virulence does not end with creating a single mutant strain. To fulfill Falkow’s molecular Koch’s postulates, and to prove any reduction in virulence is due to the deletion of the gene of interest rather than unanticipated consequences of the gene deletion process, the mutant strain must be complemented. Complementation of a gene deletion strain involves reintroducing a wild-type copy of the deleted gene back into the genome. As with the deletion process, the methodology of complementation also varies between species. In *Escherichia coli*, the reintroduction of the wild-type gene is usually straightforward, achieved via introduction of a plasmid that carries the wild-type gene of interest which is then maintained extrachromosomally [6]. In *S*. *cerevisiae*, one of three approaches is usually employed: the gene of interest is reintroduced in a high copy 2μ plasmid [7], in a low copy centromeric plasmid [8], or by integrating it into a predetermined location in the genome [9]. In *Candida albicans*, constructs can be targeted to integrate at the well characterized and highly expressed *RPS10* locus, disrupting this allele in the process [10,11]. These types of well-utilized approaches enable molecular Koch’s postulates to be easily fulfilled in the majority of organisms in which targeted gene deletions can be made.

While molecular genetic studies were originally restricted to model systems, many other organisms of interest, including pathogens, can now also be easily manipulated. One such pathogen is *Cryptococcus neoformans*, a basidiomycete yeast that primarily infects immunocompromised individuals, disseminating via the lungs to cause life-threatening meningoencephalitis. In developed countries, the mortality rate of cryptococcosis is up to 20% [12,13]. Limited treatment availability in developing countries results in a much poorer patient prognosis; without treatment the mortality rate can be as high as 100% [12,13].

In the pursuit of a deeper understanding of this pathogenic species, manipulation of the *C*. *neoformans* genome has become common, although it is not without challenges. Gene deletion via biolistic transformation in *C*. *neoformans* typically results in homologous recombination frequencies of 1–10% [14–16], far lower than in *S*. *cerevisiae*. In addition, *C*. *neoformans* is also unable to stably maintain plasmids, making complementation of mutants more difficult.

There are currently three widely used approaches for complementing a gene deletion in *C*. *neoformans*. The simplest of these is to not complement at all, with a surprising number of publications passing peer review without including this important control. Sometimes this approach is accompanied with data from a sexual cross to show the mutant phenotype is linked to the inserted selectable marker; linkage does not, however, prove that any observed phenotypes are not caused by polar effects of the selectable marker on genes adjacent to the insertion site. When complementation is performed, the most common method is to integrate a wild-type copy of the gene randomly into the genome [17,18]. However, protein-coding genes account for 85% of the *C*. *neoformans* genome, and when taking into consideration the large number of miscRNAs also transcribed [19], it is highly likely that this random integration will disrupt other genes and produce confounding phenotypes. It is possible to address this problem via the creation of multiple, independent complemented strains to find a common phenotype. Ethically, only one of these strains can be employed in a murine virulence model, making it impossible to determine whether virulence has been affected by interruption of another non-target virulence-associated gene until after infection of the animals. An alternative complementation approach is to reintroduce the wild-type gene back into its native location [20]. However, this may influence the expression of adjacent genes due to the small intergenic regions and frequently overlapping transcripts of the *C*. *neoformans* genome [19].

In this study, we further analyze the two current methodologies for complementation in *C*. *neoformans* and provide evidence of their shortcomings. To counter the drawbacks of these approaches, we have developed a new molecular tool for complementation that obviates these problems. Following an analysis of gene distribution in the genome, we have characterised a small gene-free region termed the “safe haven” and developed a vector to facilitate integration of genetic constructs at this site. We have confirmed that integration of the vector at the safe haven has no effect on the transcription of flanking genes, on a range of virulence-associated phenotypes, or on virulence itself. Beyond providing a much-needed resource to facilitate the satisfaction of Falkow’s molecular Koch’s postulates in *C*. *neoformans*, our targeted safe haven approach also provides an effective avenue for the introduction of other useful genetic constructs such as fluorescent proteins and reporter constructs.

## Methods

### Bioinformatics

The genome sequence of the *C*. *neoformans* var. *grubii* type strain H99 was obtained from the Broad Institute “*Cryptococcus neoformans* var. *grubii* H99 Sequencing Project, Broad Institute of Harvard and MIT (http://www.broadinstitute.org/)” Sequence analysis to determine the numbers of convergent, divergent and tandemly arrayed gene pairs, and the size of their intergenic regions, was performed in Excel using the relevant H99 gene file from the Broad Institute.

### Strains and growth conditions

*C*. *neoformans* was cultured in YPD (2% bacto-peptone, 2% agar, 1% yeast extract and 2% glucose) media at 30°C unless stated otherwise. Biolistic transformants were selected on YPD medium containing 100 μg/mL nourseothricin (Werner Bioagents, Germany), G418 (Sigma, USA) or hygromycin B (Life Technologies, USA). All strains (S1 Table) were stored in 15% glycerol at -80°C until use, at which point they were maintained on YPD at 4°C for a maximum of two weeks. *E*. *coli* Mach1 cells (Invitrogen, USA) served as the host strain for transformation and propagation of all plasmids using lysogeny broth supplemented with 100 μg/ml ampicillin (Sigma, USA) [21].

### Creation of a targeted integration vector

Plasmids constructed in this study are listed in S2 Table and primers in S3 Table. PCR for construct generation was performed using Phusion High Fidelity DNA Polymerase (New England Biolabs, USA). The intergenic region between H99 ORFs *CNAG_00777* and *CNAG_00778* was PCR amplified from genomic DNA as two fragments: chromosome 1 coordinates 2,045,692–2,046,441 using primers UQ2920 and UQ3107, and 2,046,485–2,047,234 using UQ2916 and UQ2917. A third fragment was amplified using UQ2918 and UQ2919 from a synthetic gBlock (IDT, USA) consisting of chromosome 1 nucleotides 2,045,692–2,045,827 followed by the restriction site-containing linker sequence GGCGCGCCACGATACTTGTGTTAATTAA and then chromosome 1 nucleotides 2,047,035–2,047,234. The three fragments were joined via overlap PCR using primers UQ2916 and UQ3107, purified, then the 1.5 kb product included in a second overlap PCR reaction using primers UQ3106 and UQ3107 to join them to the nourseothricin resistance marker *NAT* (amplified from pCH233 using primers UQ2915 and UQ3106) to give a 3.2 kb product.

pBluescript II SK- [22] was subsequently amplified with primers UQ3108 and UQ3109; both the 3.2 kb overlap and pBluescript II SK- products were then cut with NcoI and BglII, and ligated together prior to transformation into *E*. *coli*. The final product, pSDMA25, contained the safe haven, linker and nourseothricin resistance marker sequences in place of the pBluescript II SK- f1 origin of replication, maintaining the intact multicloning site and blue/white screening capability of the parent plasmid. Equivalent plasmids bearing G418 resistance (amplified from pJAF1 [23] using primers UQ2915 and UQ3106 to give pSDMA57) or hygromycin resistance (amplified from pJAF15 using primers UQ2915 and UQ3106 to give pSDMA58) markers were generated using the same strategy.

### Construction of mutant strains

A deletion construct for the *ADE2* gene was generated using overlap PCR, joining the *ADE2* 5’ region (primers UQ1439 and UQ1440), the G418 resistance marker *NEO* (UQ234 and UQ235) and *ADE2* 3’ region (UQ1441 and UQ1442); H99 genomic DNA was used as the template for *ADE2* and pJAF1 for *NEO* [23]. *C*. *neoformans* transformation was carried out via biolistic particle delivery as previously described onto media containing G418 [24]. The *ade2*Δ strain SA26 was identified via its pink coloration and adenine auxotrophy, with correct integration confirmed by Southern blot.

To create wild-type strains carrying the empty safe haven vector, pSDMA25 was linearized with either AscI, BaeI or PacI as indicated and biolistically transformed into the recipient strain; to create an *ade2*Δ strain carrying the empty vector, BaeI was used. To complement the *ade2*Δ mutant using random integration, ADE2 was PCR amplified (UQ1439 and UQ1442) and cloned as a HindIII/EcoRI fragment into HindIII/EcoRI cut pBluescript II SK- to give pSDMA42. *ADE2* was subsequently subcloned as an XbaI/XhoI fragment into XbaI/XhoI-cut *NAT* resistance vector pCH233 to create pSDMA55. pSDMA55 was then linearized with PvuII and biolistically transformed into the *ade2*Δ strain SA26. To complement the *ade2*Δ mutant by reintroducing the gene at the wild-type locus, *ADE2* (UQ1439 and UQ3342), *NAT* (UQ3343 and UQ3344) and the *ADE2* 3’ region (UQ3345 and UQ3243) were amplified then joined via overlap PCR, with the product transformed into the *ade2*Δ strain SA26. To complement the *ade2*Δ mutant using targeted safe haven integration, ADE2 was subcloned from pSDMA42 as a SacII/XhoI fragment into SacII/XhoI-cut pSDMA25 to create pSDMA54. pSDMA54 was subsequently linearized using AscI and biolistically transformed into the *ade2*Δ strain SA26. All transformants were selected on YPD supplemented with nourseothricin, and confirmed by Southern blot analysis.

### Multiplex colony PCR

Multiplex PCR was employed to confirm correct integration of the safe haven-targeting constructs. 12 μL of sterile water and a small amount of *Cryptococcus* cells on the end of a pipette tip were added to a PCR tube for each reaction and incubated for 10 minutes at 94°C. 2.5 μL of each of the 10 mM primer stocks (UQ1768, UQ2962, UQ2963 and UQ3348), 2.5 μL Taq buffer, 0.5 μL 10 mM dNTPs and 0.1 μL of Taq polymerase (New England Biolabs, USA) were then added and the reaction returned to the PCR machine. The cycling parameters were 35 cycles of 94°C for 20 seconds, 55°C for 20 seconds and 72°C for 90 seconds. Products were visualized using electrophoresis with a 1% TAE agarose gel.

### Quantitative real-time PCR

*C*. *neoformans* strains were grown in YNB with shaking at 30°C for 16 hours. Cultures were harvested, cell pellets frozen and lyophilized, and total RNA isolated using TRIzol reagent (Life Technologies, USA). cDNA was generated using the Superscript III First-Strand Synthesis System (Invitrogen, USA). Primers for *ADE2* (*CNAG_02294*), the genes flanking *ADE2* (*CNAG_02293* and *CNAG_02295*) and the genes flanking the safe haven insertion site (*CNAG_00777* and *CNAG_00778*) were designed to span exon–exon boundaries. Quantitative real-time PCR (qRT-PCR) was performed using SYBR Green Supermix (Applied Biosystems) and an Applied Biosystems 7900HT Fast Real Time PCR System with the following cycling conditions: denaturation at 95°C for 10 minutes, followed by amplification and quantification in 45 cycles of 95°C for 15 seconds and 60°C for 1 minute, with melting-curve profiling at 95°C for 2 minutes, 60°C for 15 seconds, and 95°C for 15 seconds. Relative gene expression was quantified using SDS 1.3.1 (Applied Biosystems) based on the 2^-ΔΔCT^ method [25]. The housekeeping actin-encoding gene *ACT1* was used as a control for normalization. One-way analysis of variance was performed using the unpaired, two-tailed *t*-test in GraphPad Prism Version 6.0c (GraphPad Software, USA). *P*-values of <0.05 were considered statistically significant.

### Murine inhalation model of cryptococcosis

For murine infection assays, 6-week-old female BALB/c mice were infected via nasal inhalation [26]. For each strain, 10 mice were inoculated with a 50 μL drop containing 5 × 10^5^ cryptococcal cells. Mice were weighed daily, and once their body weight had decreased to 80% of pre-infection weight (or on the final day of the experiment), were euthanized using CO_2_ inhalation. For survival assays, Kaplan-Meier survival curves were plotted using GraphPad Prism 6.0 (GraphPad Software, USA). Significance was tested by using Students *t*-test on the logged data. *P*-values of <0.05 were considered significant.

### Ethics statement

This study was carried out in strict accordance with the recommendations in the Australian Code of Practice for the Care and Use of Animals for Scientific Purposes by the National Health and Medical Research Council. The protocol was approved by the Molecular Biosciences Animal Ethics Committee (AEC) of The University of Queensland (AEC approval no. SCMB/439/13/UQ/NHMRC). Infection was performed under methoxyflurane anesthesia, and all efforts were made to minimize suffering through adherence to the Guidelines to Promote the Wellbeing of Animals Used for Scientific Purposes as put forward by the National Health and Medical Research Council (Australia).

## Results

### Identifying locations in the *Cryptococcus* genome suitable for construct integration

Given it is an essential part of molecular genetic studies in *C*. *neoformans*, the creation of complemented derivatives of mutant strains has long proven a surprisingly problematic process. The sequencing of two *C*. *neoformans* var. *neoformans* strains revealed the genome of this species to be very gene-rich [19]. Consequently, reintroduction of a wild-type copy of the gene of interest through random integration is essentially a round of insertional mutagenesis which will more than likely disrupt another gene in the process, explaining the frequent phenotypic defects identified in complemented *C*. *neoformans* strains. Reinsertion at the original locus is no better, potentially leaving the new strain indistinguishable from a wild-type contaminant, and abolishing polar effects (or introducing new ones) on adjacent genes that may have contributed to the original mutant phenotype.

To provide a controlled, consistent complementation process we searched the genome for potential locations to which we could target an integrating vector without influencing other genetic factors. Our first consideration regarding suitability of a defined insertion site was gene distribution. Ideally, we wanted to find a gene-poor region of the genome to target our construct. One option was to target one of the large transposon fragment-rich centromeres, which are devoid of genes [19,27]. As the transcriptional fate of a construct targeted to such a region is unknown and highly likely different to wild-type, and as the repeat-rich nature of the region would make targeting difficult, we chose to instead identify a location elsewhere in the genome. Unfortunately, our previous analysis of gene distribution revealed that *C*. *neoformans* has a consistent distribution of genes across the genome, unlike species such as *S*. *cerevisiae* that exhibit reduced gene density in subtelomeric regions [28]. As no obvious gene-poor regions exist, it became necessary to systematically analyze all intergenic regions in the genome to identify the best candidate location.

### Selecting a safe haven site in the *C*. *neoformans* genome

Our criteria for identifying a potential safe haven site for insertion of genetic constructs were twofold. First, the safe haven must be flanked by convergently transcribed genes; as no promoters in *C*. *neoformans* have been exhaustively characterized, we could not risk putting our constructs in a potential promoter region and disrupting transcription of a downstream gene. Second, the safe haven must be one of the larger intergenic regions in the genome to minimize the chance of inserted constructs influencing adjacent genes.

**Fig 1:**
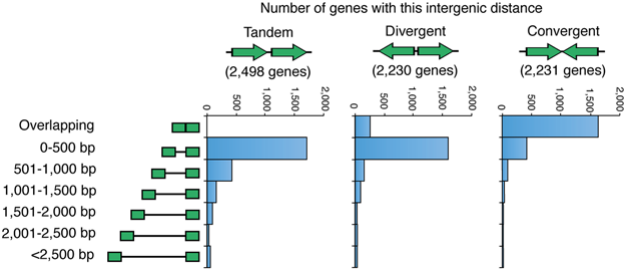
Gene distribution and spacing in the *Cryptococcal* genome. Distribution of intergenic distances in *C*. *neoformans* with the frequency of neighboring genes.

Of the almost 7,000 predicted protein-coding genes identified via the recently completed H99 genome project [17], roughly one third of gene pairs are tandemly transcribed, one third divergently transcribed, and one third convergently transcribed (Fig 1). Consistent with the compact nature of most characterized fungal promoters, tandemly and divergently transcribed genes in *C*. *neoformans* tend not to overlap. Furthermore, the inter-transcript distance is usually short, with 68% of tandem and 71% of divergent inter-transcript distances less than 500 bp. Inserting a construct within one of these small inter-transcript regions is likely to influence a promoter, making them unsuitable targets. In contrast, almost 73% of the 2,231 convergently transcribed gene pairs have overlapping transcripts (Fig 1); while these do not contain promoters, they still cannot be used as a potential insertion site without disrupting a known transcript.

Of the 587 intergenic regions left between convergently transcribed genes with non-overlapping transcripts, the majority were deemed unsuitable as candidate insertion sites due to their small size; 418 had an inter-transcript distance of less than 500 bp. To minimize the probability of our inserted constructs affecting adjacent genes we decided to choose a site that was larger. However, as the H99 genome has on average one gene every 2,636 bp (when the centromeres are excluded), we also wanted to limit the upper size of the region to reduce the possibility of it containing an uncharacterized gene or transcript. We therefore chose to focus on the intergenic regions between convergently transcribed genes that had an inter-transcript distance between 1,500–2,000 bp in length. Only 18 intergenic regions met these criteria, and comparison of these regions to the extensive transcriptome data generated by Christine Cuomo, Guilhem Janbon and colleagues revealed that 12 contain miscRNAs of unknown function, making them unsuitable as a safe haven site [19]. Of the remaining 6 intergenic regions, we chose the smallest, reasoning that it should be less likely to contain an unidentified gene. This 1,544 bp intergenic region is found on chromosome 1 between the convergently transcribed *CNAG_00777* and *CNAG_00778* genes.

### Creating an integration vector that targets the safe haven site

**Fig 2:**
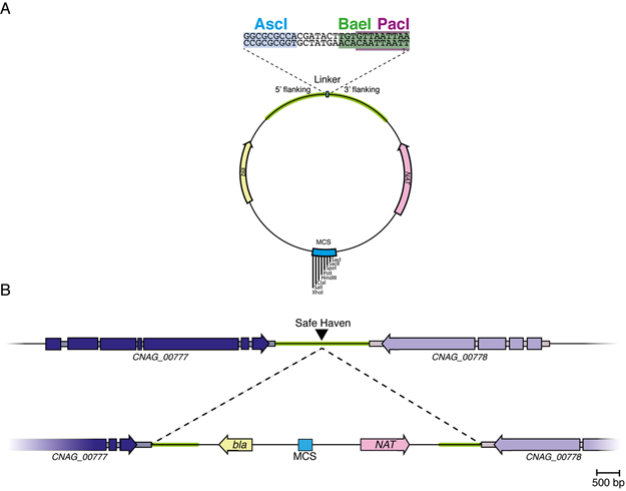
Targeted integration vector. *A*. The vector created has several key features. These include an intact multicloning site and blue-white screening for cloning the genetic construct of interest, a dominant selectable marker (here *NAT*, but also constructed in *NEO* and *HYG* versions), *bla*, a bacterial selection marker, and 5’ and 3’ flanking regions that are homologous to the safe haven site in the genome. The polylinker sequence contains three rare cutting restriction enzyme recognition sites that are used to linearize the vector so that the 5’ and 3’ regions are subsequently flanking the construct. *B*. Representation of the selected safe haven site, and how the linearized vector inserts via homologous recombination.

After selecting a safe haven site, we next designed a series of targeting vectors to facilitate integration of constructs of interest at this location. The three vectors created each contain the safe haven site sequence in the backbone of pBluescript II SK- to enable integration into the *C*. *neoformans* genome via homologous recombination, in addition to a linker sequence containing multiple rare-cutting restriction enzyme cut sites to facilitate linearization prior to biolistic transformation. Each vector also includes either the *NAT* (pSDMA25), *NEO* (pSDMA57) or *HYG* (pSDMA58) dominant selectable marker (Fig 2A). Importantly, as the safe haven and marker fragments were inserted into the pBluescript II SK- backbone in place of the f1 origin of replication, each plasmid still retains the original multicloning site and blue/white screening capabilities (Fig 2A).

Linearization of the safe haven constructs is made possible by the inclusion of a linker containing cut sites for three rare cutting restriction enzymes—BaeI, AscI and PacI (Fig 2A). To use the vectors, the gene of interest is subcloned into the standard pBluescript II SK- multicloning site and sequenced to ensure it is error free. Subsequently, the vector is cut using either BaeI, AscI or PacI, a choice influenced by the sequence of the fragment that has been cloned into the multicloning site. Following linearization of the plasmid, the safe haven homology regions flanking the gene of interest facilitate homologous recombination at the safe haven site following biolistic transformation (Fig 2B).

### Multiplex PCR reveals small non-homologous overlaps slightly inhibit integration rates

**Fig 3:**
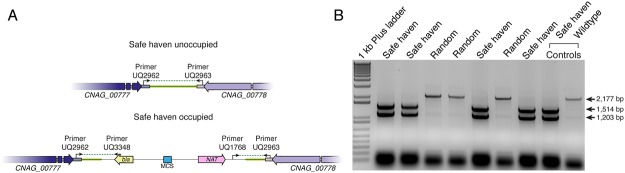
Multiplex PCR for screening successful genomic integration events. *A*. Depiction of the genome with the primers used in the multiplex PCR. *B*. Representative multiplex colony PCR results from transformants of H99 with the empty vector cut with BaeI. Transformants yielding two bands indicate the construct has integrated correctly, while incorrect transformants only have one band.

To simplify preliminary identification of transformants carrying the targeting vector at the desired safe haven location, we designed a diagnostic multiplex PCR (Fig 3A). The two outer primers yield a single band (2,177 bp) when a colony does not carry the safe haven vector at the correct location (Fig 3B). However, when the construct successfully integrates at the safe haven site, the additional primer binding sites introduced by the construct permit the amplification of two products (1,514 bp and 1,203 bp). In the event of tandem integration of the construct at the safe haven site, a 2,157 bp band would also be evident along with the 1,514 bp and 1,203 bp fragments, however we never observed this event.

While a simple PCR analysis is a convenient method to rapidly screen transformants, it does not discriminate between strains that may carry additional, ectopic copies of the vector. To ensure that our transformants carry only a single copy of our vector, and that copy was at the safe haven site, we always perform Southern blot analysis prior to the strain being used for further work. Of 76 positive PCR colonies tested in this way so far, only 13 were incorrect as identified by Southern blot.

**Fig 4:**
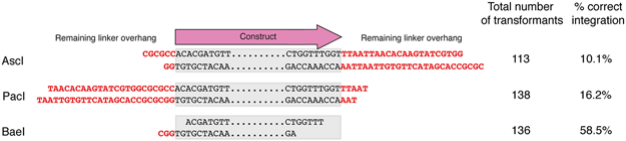
Non-homologous ends inhibit successful homologous integration. Illustration of the polylinker sequence after being cut with each of three rare cutting restriction enzymes. The construct depiction indicates where the marker and gene as well as the two flanking regions would be located after linearization. The red sequence shows residual polylinker sequence after digestion. Percent integration indicates the proportion of antibiotic resistant colonies in which the targeting vector was correctly integrated at the safe haven site as determined by multiplex PCR after biolistic transformation.

Due to the necessary inclusion of a linker sequence in the targeting vector, a small stretch of this sequence remains on the ends of the product following linearization—sequence that differs from the safe haven flanking sequence on chromosome 1. Cutting with PacI or AscI leaves up to 26 bp of non-homologous sequence, while BaeI digestion leaves only 3 bp of overhang, of which 2 bp are identical to the genome sequence. To determine whether these small non-homologous ends inhibit correct integration of a construct we performed a series of transformations of wild-type strain H99, integrating the empty pSDMA25 safe haven construct linearized with each of the linker-cutting enzymes. PacI- and AscI-digested vector incorporated at the correct position in 16% and 10% of resistant transformants, respectively. These frequencies are towards the upper end of published *C*. *neoformans* rates of homologous integration [14–16]. In contrast, BaeI-digested vector incorporated at the correct location in over 58% of resistant transformants, much higher than is typically observed (Fig 4). Together, these data indicate that while AscI and PacI digestion yield excellent homologous integration rates of the vector by current standards in *C*. *neoformans*, BaeI is the restriction enzyme of choice when no BaeI site exists in the genetic construct cloned into the safe haven vector.

### Integration of the targeted vector does not affect the expression of genes flanking the safe haven site, or alter virulence factor-related phenotypes

**Fig 5:**
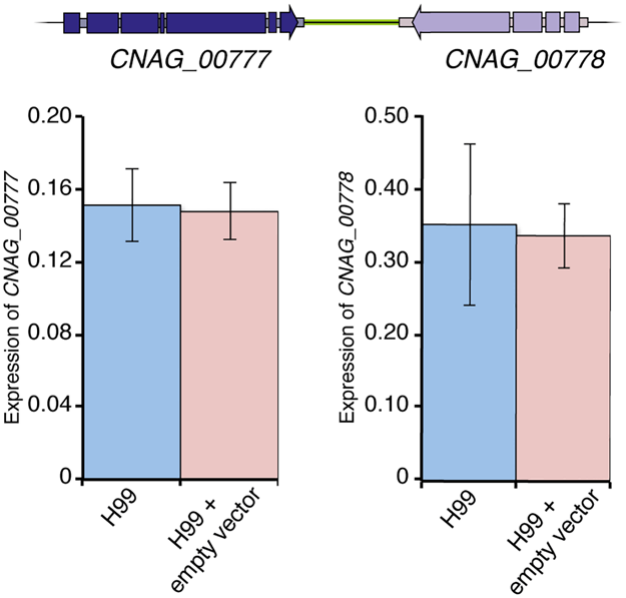
Integration of the vector into the safe haven site does not affect expression of flanking genes. Transcript abundance of *CNAG_00777* and *CNAG_00778* relative to *ACT1* with (H99 + empty vector) and without (H99) integration of vector at the safe haven site. Values show mean, error bars show S.E.M.

To confirm that insertion of the targeting vector did not affect expression of the two genes flanking the safe haven site (*CNAG_00777* and *CNAG_00778*), qRT-PCR was performed using total RNA extracted from both strain H99 and an H99 transformant carrying the correctly integrated safe haven vector. Under standard growth conditions, there was no significant difference in the abundance of either *CNAG_00777* or *CNAG_00778* transcripts when comparing wild-type H99 to the H99 transformant carrying the empty safe haven vector (Fig 5). Furthermore, we found no observable change in the production of the key virulence factors urease, phospholipase, protease and melanin in the strain carrying the safe haven vector, nor was there a change in growth rate at either 30°C or 37°C (data not shown). These results further validate the suitability of the chosen site for construct targeting.

### Employing an *ade2*Δ mutant to test the various approaches to complementation

The purpose of creating the targeting vectors was to ensure we could efficiently and reproducibly complement mutant strains without introducing unwanted secondary mutations. To verify that the targeting vector functioned as desired, we first deleted the well-characterized phosphoribosylaminoimidazole carboxylase-encoding *ADE2* gene [29]. Loss of *ADE2* results in the easily observable phenotypes of mutant colonies turning pink due to accumulation of the purine biosynthetic intermediate P-ribosylaminoimidazole, as well as adenine auxotrophy [30].

**Fig 6:**
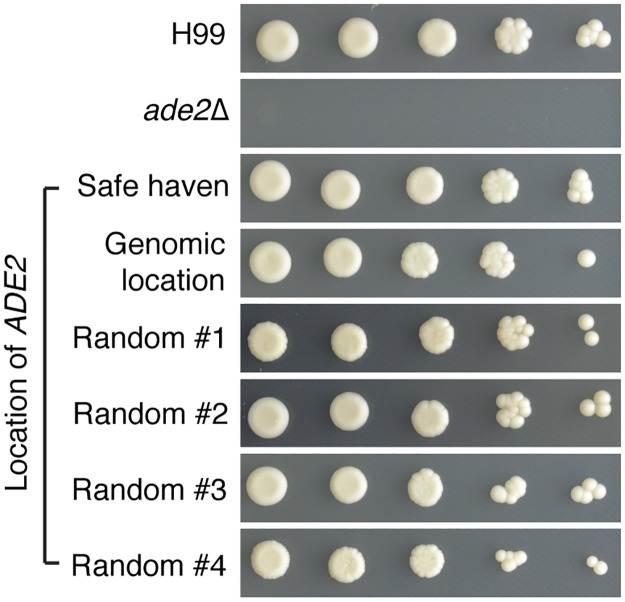
An *ade2*Δ mutant cannot grow on YNB media lacking adenine. All complemented strains created in this study have wild-type levels of growth on YNB media and are of normal color.

**Fig 7:**
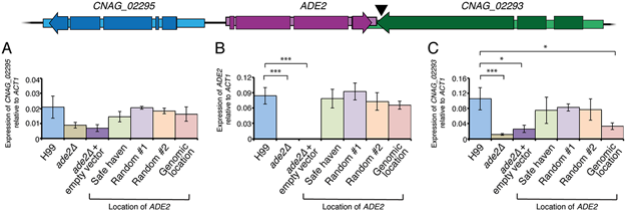
Expression of *ADE2* and flanking genes in an *ade2*Δ strain complemented with targeted or random integration. Expression of (*A*) *CNAG_02295*, (*B*) *ADE2* and (*C*) *CNAG_02293* relative to *ACT1* in various strains. The black arrow indicates where the *NAT* selectable marker was inserted to complement in the strain “Genomic Location”. Values show mean, error bars show S.E.M. * = P<0.05; *** = P<0.01.

To compare our new targeting method to established complementation protocols we complemented the *ade2*Δ mutant using three different approaches. First, we used random integration by transforming the *ade2*Δ mutant with a linearized plasmid carrying wild-type *ADE2* and the *NAT* selectable marker, but no targeting sequence. As independent random integrants will each have the wild-type *ADE2* gene/nourseothricin-resistance cassette inserted at a different site in the genome, we selected four transformants for initial analysis. After observing that all four strains complemented the *ade2*Δ strain’s mutant phenotypes, two were randomly selected for further analysis: strains “Random #1” and “Random #2”. Second, we reinserted the wild-type gene at the original locus by targeting integration at the *ade2*Δ allele with a construct consisting of *ADE2* plus the *NAT* marker at the 3’ end; this strain was named “Genomic Location”. Finally, the *ade2*Δ mutant was also complemented using targeted integration at the safe haven site to produce the strain “Safe Haven”. All three strategies complemented the *ade2*Δ mutant’s adenine auxotrophy and pink pigmentation (Fig 6). Furthermore, qRT-PCR confirmed that all three methods of complementation returned *ADE2* expression levels to wild-type (Fig 7).

### Complementation of *ade2*Δ using the safe haven strategy fully restores virulence, but random integration or targeting to the genomic locus does not

**Fig 8:**
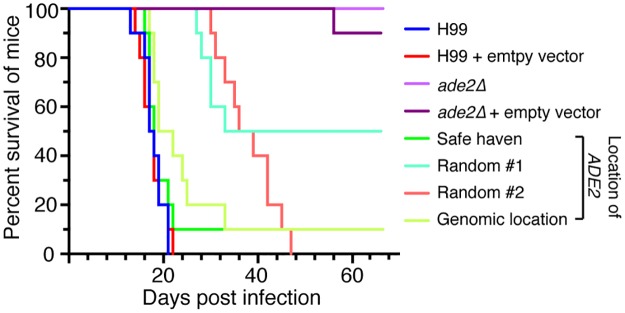
Virulence in a mouse model. *A*. No significant difference was found between H99, H99 carrying the empty targeting plasmid, and the *ade2*Δ strain complemented with *ADE2* using the safe haven targeting plasmid. While the *ade2*Δ mutant and the *ade2*Δ strain carrying the empty safe haven showed no significant difference from each other, a significant difference in survival was seen between each and H99 (*P*<0.0001). Significant differences were also observed between H99 and complemented strains Random #1, Random #2 and Genomic Location (*P*<0.0001, *P*<0.0001 & *P*<0.05 respectively).

To compare the robustness of the three different approaches to complementation, we conducted virulence assays in a murine inhalation model using all four *ade2*Δ complemented strains—the two random integrants, the genomic location strain, and the safe haven strain. We tested these alongside wild-type and *ade2*Δ controls, as well as derivatives of these controls that carried the safe haven vector (Fig 8).

As previously reported [29], the *ade2*Δ mutant was severely impaired for virulence compared to the wild-type strain. A crucial observation for this study was that the wild-type and *ade2*Δ controls carrying the empty safe haven vector exhibited equivalent virulence to their parental strains; that is, inserting the targeting vector at the safe haven site did not influence virulence in either the virulent wild-type strain, or the attenuated *ade2*Δ mutant.

Interestingly, the four complementation strains all exhibited differing survival times in the mouse model, despite our qRT-PCR results showing that each expressed *ADE2* at wild-type levels under our tested condition. The *ADE2* random integrants both partially restored virulence, but poorly. The strain in which the *ADE2-NAT* cassette had been targeted to the original *ADE2* genomic location exhibited virulence more closely resembling wild-type, but was still statistically different. In contrast, the complemented strain carrying *ADE2* at the safe haven site showed no statistical difference to wild-type, indicating a complete restoration of virulence. Of the three strategies employed for complementation, the safe haven approach was the only one that restored virulence to a level indistinguishable from wild-type, confirming that our safe haven approach was the superior complementation strategy.

### The hazards of complementation via random integration or targeting the original genomic locus

We wanted to determine why the non-safe haven complementation strains failed to restore virulence to a wild-type level. Beginning with random integration strains Random #1 and Random #2, we employed an inverse PCR approach to identify the insertion sites of the *ADE2/NAT* vector in the genome of these strains. Sequencing of PCR products obtained revealed that, as could be expected, each disrupted another gene. In strain Random #1 the *ADE2/NAT* random integration vector had inserted into exon 4 of *CNAG_06080* on chromosome 12, a gene whose product exhibits 48% similarity to the *S*. *cerevisiae* phosphatidylinositol phosphate phosphatase Sac1. Unsurprisingly, like the *S*. *cerevisiae sac1*Δ mutant, the Random #1 strain is an inositol auxotroph, and this likely explains the compromised virulence of this strain. In strain Random #2 the *ADE2/NAT* random integration vector had inserted 9 bp upstream of the start point of transcription of *CNAG_05028* on chromosome 4, a gene whose product exhibits 34% similarity to the *S*. *cerevisiae* cysteine synthase Cys4. Again, just as the *S*. *cerevisiae cys4*Δ mutant is a cysteine auxotroph, so is strain Random #2, and this auxotrophy likely explains the compromised virulence. Importantly, neither of these auxotrophies are observable on either YPD or YNB, the two standard media that are used almost exclusively for routine cultivation of *C*. *neoformans* in the laboratory setting and which both contain inositol and cysteine.

Analysis of the Genomic Location complementation strain revealed a different type of problem. Our qRT-PCR results revealed that the *ade2*Δ mutant has a broad effect on the expression of all of the genes we investigated with this technique (S1 Fig). While this observation has not been reported before, we found it unsurprising; as Ade2 is required for *de novo* ATP and GTP synthesis, it is necessary for the production of two of the four nucleotides required for RNA synthesis. Complementation of the *ade2*Δ mutation using the safe haven approach or random integration resolves this phenotype. However, the Genomic Location strain does not return to normal expression levels for *CNAG_02293*. To ensure the entire wild-type *ADE2* transcript was produced, the complementation construct we generated to create the Genomic Location strain included the *NAT* selectable marker slighty after the *ADE2* transcriptional endpoint. The *CNAG_02293* gene downstream of *ADE2* is convergently transcribed, and like the majority of convergently transcribed genes in this species, the *CNAG_02293* and *ADE2* transcripts overlap. The consequence of this overlap is that our insertion of the *NAT* marker actually occurs in the 3’ end of the *CNAG_02293* coding region, truncating the predicted 837 aa product by 85 residues. Furthermore, our qRT-PCR data shows that in this strain, *CNAG_02293* transcript is significantly less abundant than in wild-type. As this gene is not a clear homolog of any well-characterised gene in another species, we cannot predict the biochemical reason underpinning why the Genomic Location strain exhibits a slightly, but still statistically significant, reduction in virulence.

## Discussion

The current molecular tools and methodologies available for the study of *C*. *neoformans* are amongst the best in fungal species, making it not only a readily tractable pathogen but also a valid model system for the study of fundamental biological processes. However while gene deletions are relatively simple to create via techniques used widely within the *C*. *neoformans* research community, the approach to the equally important step of complementation is less well prescribed.

In this study we have analyzed the two complementation methods currently accepted by the field and explored their shortcomings. Results confirm that of the two methods, complementing via random integration shows the greatest potential for problems because it will likely interrupt other genes. As the vast majority of the *C*. *neoformans* genome is transcribed, the chance of the complementation construct disrupting a gene or promoter region is high. We had reasoned that because *ADE2* expression in our random integration strains was equivalent to wild-type, that they should behave as wild-type in a murine inhalation model. This assumption was incorrect; both strains carried conditional lethal mutations that were not obvious using standard laboratory growth conditions, but were apparent upon infection of mice. To our knowledge, this study is the first to characterize insertion sites of complementation constructs in *C*. *neoformans*, despite the approach of random integration being the basis of insertional mutagenesis performed by others in this same species [31]. Our observations show that if a random insertion approach is employed, then care should be taken to identify the exact location of insertion of the complementation construct prior to animal experiments for ethical reasons to reduce the probability that secondary virulence defects are observed, resulting in unpublishable infection data.

The more conservative complementation approach employed by the community is reintroducing the wild-type gene along with a dominant selectable marker at the original genomic location. We also encountered problems with this strategy, with the highly compressed genome, short intergenic regions and overlapping transcripts of the organism resulting in our example of this strategy compromising the downstream gene. Retrospectively, we now know that the problems we encountered could have been avoided with the aid of the recently released transcriptome information by incorporating appropriate duplicated sequence in our insertion construct. While this information was not available when the strain was originally generated, this example does highlight the complexities that must be considered when retargeting the gene of interest to its original genomic location.

The major factor we needed to consider in this study was identifying an appropriate site in the *Cryptococcus* genome to target genetic constructs. In *S*. *cerevisiae* auxotrophy markers are widely utilized, and loci such as *LEU2*, *URA3* and *HIS3* serve as excellent locations for the insertion of genetic constructs. However in species where pathogenicity is being studied these same makers can be problematic due to the nutritional environment encountered in the infected animal. When auxotrophic and prototrophic strain pairs were assessed in a murine model in *C*. *albicans*, it was found that mutants auxotrophic for uracil, adenine and heme each showed a lower level of pathogenicity relative to control strains [32]. While these shortcomings can be overcome by targeting constructs to the highly expressed *RPS10* locus [11,33], this example highlights the inherent risks associated with the use of auxotrophic markers in the study of pathogens. It is therefore unsurprising that while auxotrophic markers have been developed in *C*. *neoformans*, they are not widely used [34].

Here we characterized a safe haven site to be used for targeted integration of complementation constructs in *C*. *neoformans* and propose a strategy that has distinct advantages over current approaches. The safe haven strategy we employed is much simpler and more straightforward than existing methods of complementation. Our method allows for easy cloning, transformation and identification of positive inserts of a construct. It overcomes the current problems of influencing the expression of neighbouring genes, or disrupting them outright. We have successfully employed it to complement multiple mutants beyond those described in this manuscript, as well as to introduce tagged proteins and genes from other species. In all cases, the transformants have behaved exactly as we would expect. Based on this success, the use of the safe haven approach for introduction of genetic constructs has not only become the default approach in our own laboratory, but also in the laboratories of a number of our colleagues in the *C*. *neoformans* community. In short, we have designed a new methodology that we believe is the best option for complementing deletion strains and introducing genetic constructs into the genome of *C*. *neoformans*.

## Supporting Information

S1 Fig Expression of *ade2*Δ and *ade2*Δ containing the empty vector. Expression of *CNAG_02295*, *CNAG_02293*, *CNAG00077* and *CNAG00078* relative to *ACT1* in various strains. Values show mean, error bars show S.E.M. * = P<0.05; *** = P<0.01.
(TIF)

S1 Table Strains used in this study. (DOCX)

S2 Table Plasmids used in this study. (DOCX)

S3 Table Primers used in this study. (DOCX)
