# Supplementary figures and images for: A Genomic Safe Haven for Mutant Complementation in Cryptococcus neoformans
Source: PLoS One. 2015 Apr 9;10(4):e0122916. doi: 10.1371/journal.pone.0122916 (PMC4391909; doi:10.1371/journal.pone.0122916)

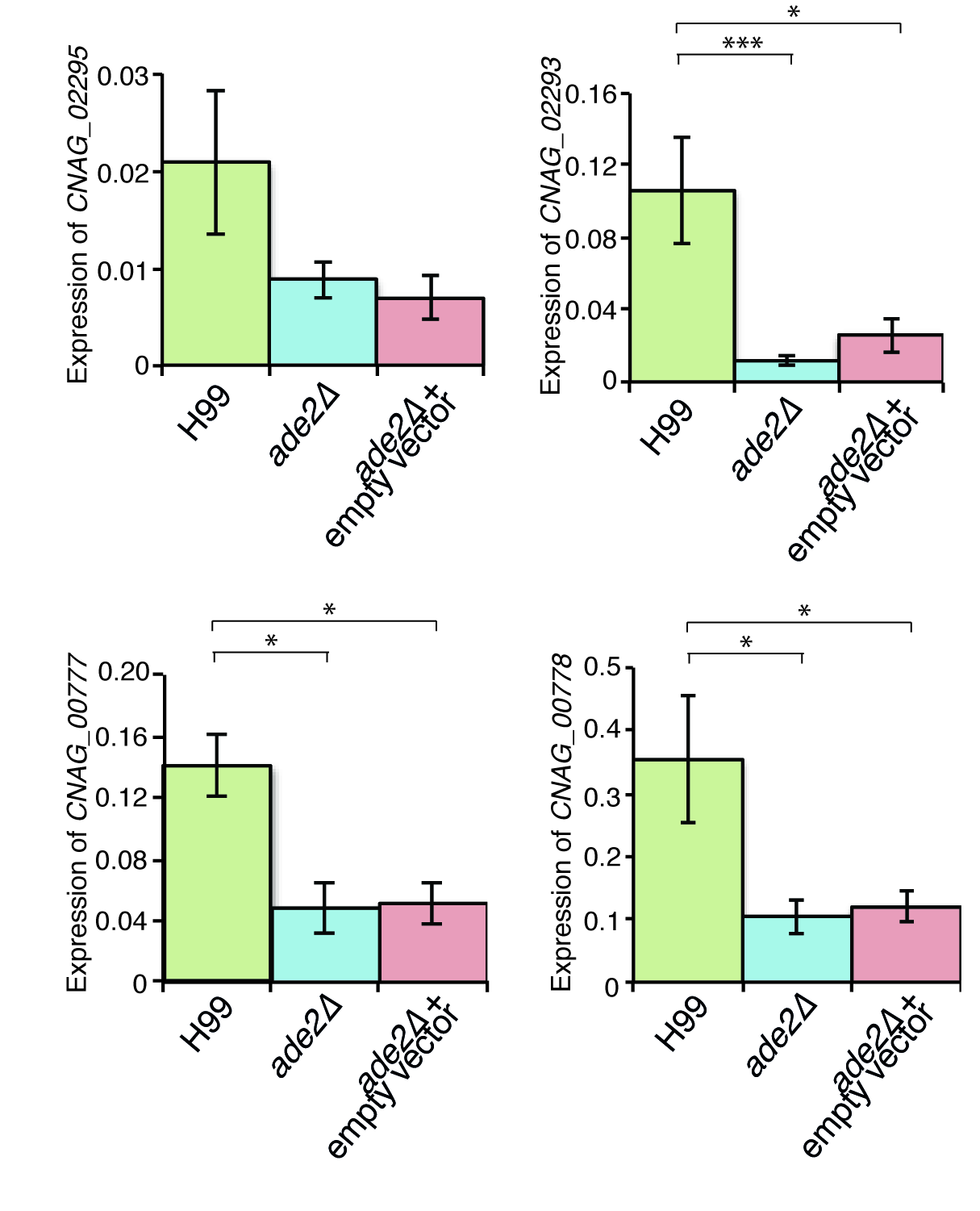

Supplement: S1 Fig — Expression of CNAG_02295, CNAG_02293, CNAG00077 and CNAG00078 relative to ACT1 in various strains. Values show mean, error bars show S.E.M. * = P<0.05; *** = P<0.01. (TIF) [file pone.0122916.s001.tif]
