# Supplementary material for: A Genomic Safe Haven for Mutant Complementation in Cryptococcus neoformans
Source: PLoS One. 2015 Apr 9;10(4):e0122916. doi: 10.1371/journal.pone.0122916 (PMC4391909; doi:10.1371/journal.pone.0122916)
Supplement: S1 Table — (DOCX) [file pone.0122916.s002.docx]

**Supplementary Table 1: Fungal strains used in this study**

| Strain | Genotype | Original source |
| --- | --- | --- |
| H99 | *Cryptococcus neoformans* MATα | [36] |
| SA22 | H99 + empty safe haven integrant | This study |
| SA26 | *ade2::NEO* MATα | This study |
| SA39 | *ade2::NEO* + empty safe haven integrant | This study |
| SA53 | *ade2::NEO* + *ADE2::NAT* (Safe haven) | This study |
| SA54 | *ade2::NEO* + *ADE2::NAT* (Genomic location) | This study |
| SA43 | *ade2::NEO* + *ADE2::NAT* (Random # 1) | This study |
| SA46 | *ade2::NEO* + *ADE2::NAT* (Random # 2) | This study |
| SA41 | *ade2::NEO* + *ADE2::NAT* (Random # 3) | This study |
| SA55 | *ade2::NEO* + *ADE2::NAT* (Random # 4) | This study |
|  |  |  |
|  |  |  |
