# Supplementary material for: A Genomic Safe Haven for Mutant Complementation in Cryptococcus neoformans
Source: PLoS One. 2015 Apr 9;10(4):e0122916. doi: 10.1371/journal.pone.0122916 (PMC4391909; doi:10.1371/journal.pone.0122916)
Supplement: S2 Table — (DOCX) [file pone.0122916.s003.docx]

**Supplementary Table 2: Plasmids used in this study**

| Strain | Genotype | Original source |
| --- | --- | --- |
| pBLUESCRIPT-SK | phagemid vector | [22] |
| pSDMA25 | NAT vector – targeted integration | This study |
| pCH233 | NAT vector – random integration | Unpublished |
| pSDMA54 | *ADE2* in pSDMA25 | This study |
| pSMDA55 | *ADE2* in pCH233 | This study |
| pSDMA57 | NEO vector – targeted integration | This study |
| pSDMA58 | HYG vector – targeted integration | This study |
| pJAF1 | NEO vector | [23] |
