# Supplementary material for: A Genomic Safe Haven for Mutant Complementation in Cryptococcus neoformans
Source: PLoS One. 2015 Apr 9;10(4):e0122916. doi: 10.1371/journal.pone.0122916 (PMC4391909; doi:10.1371/journal.pone.0122916)
Supplement: S3 Table — (DOCX) [file pone.0122916.s004.docx]

**Supplementary Table 3: Primers used in this study**

| Primer ID | NAME | Sequence |
| --- | --- | --- |
| UQ234 | NAT and linker overlap sequencing primer | GCTGCGAGGATGTGAGCTGGAGAGCG |
| UQ235 | NAT and linker overlap sequencing primer | GGTTTATCTGTATTAACACGGAAGAGATGTAG |
| UQ1438 | NAT and linker overlap sequencing primer | AACTCCGTCGCGAGCCCCATCAAC |
| UQ1439 | *ADE2* deletion construct | GAGTTAAAGTGTCGATGGCAG |
| UQ1440 | *ADE2* deletion construct | CCAGCTCACATCCTCGCAGCTTTGCTACAAGGGGTGCGGATG |
| UQ1441 | *ADE2* deletion construct | CCGTGTTAATACAGATAAACCATTGGTGCGATATCTGTAACT |
| UQ1442 | *ADE2* deletion construct | CGCTTAGGACAAGAGAGGCTA |
| UQ1696 | NAT and linker overlap sequencing primer | GGTGAAGATTGGGGAGGACAA |
| UQ1768 | Multiplex PCR primer | TCAGCAACGCCGTTGAATCCT |
| UQ2915 | NAT and linker overlap sequencing primer | TTGAGCGTGCTTCATTGGCCGGTTTATCTGTATTAACACG |
| UQ2916 | NAT and linker overlap sequencing primer | CGTGTTAATACAGATAAACCGGCCAATGAAGCACGCTCAA |
| UQ2917 | NAT and linker overlap sequencing primer | ACAATGGAATATTTCGCCTTGCCTTGGATCTTTGGCCTCT |
| UQ2924 | Bluescript sequencing primer 1 | GTGAGCAAAAGGCCAGCAAAA |
| UQ2925 | Bluescript sequencing primer 2 | CCAATGCTTAATCAGTGAGGC |
| UQ2926 | Bluescript sequencing primer 3 | GTAAACTCGCCCAACATGTCT |
| UQ2962 | Multiplex PCR primer | GGGTATGCCACAGATGCAGAT |
| UQ2963 | Multiplex PCR primer | TTGGATCCTCAATTGTCTCCT |
| UQ3106 | NAT overlap primer with NcoI site | ACGCACACCATGGCTGCGAGGATGTGAGCTGGA |
| UQ3107 | Linker overlap primer with BgIII site | TCATCTAGATCTGCCCATAGACTTCAAATCATG |
| UQ3108 | Bluescript primer with Ncol site | ACGCACACCATGGACTCAACCCTATCTCGGTCT |
| UQ3109 | Bluescript primer with BgIII site | CTCATCAGATCTTGCCGCTACAGGGCGCGTCAG |
| UQ3255 | *ADE2* sequencing primer | GCTCCTTCACGCCCGCCAAAC |
| UQ3256 | *ADE2* sequencing primer | AGATCACAATTTGCGACGACA |
| UQ3257 | *ADE2* sequencing primer | TGGGCACGACTGTCATTGGCA |
| UQ3258 | *ADE2* sequencing primer | CAGTGTACTGGACGGCGTGGA |
| UQ3265 | *CNAG_00777* qRT-PCR | GGGAGCGCGACAAATGACT |
| UQ3266 | *CNAG_00777* qRT-PCR | GACCACATCGACAGAGAAATGC |
| UQ3267 | *CNAG_00778* qRT-PCR | GACTGACGCACTATGCCAACTT |
| UQ3268 | *CNAG_00778* qRT-PCR | CCGGCAAGAGAAGGTTGAAC |
| UQ3278 | *ADE2* qRT-PCR | TGCAAATGCCCCGAGGTAT |
| UQ3279 | *ADE2* qRT-PCR | GGGCGGCATTTGTAGAGTTG |
| UQ3338 | *CNAG_02293* qRT-PCR | CGGAGAATCCAACTCTGAACCT |
| UQ3339 | *CNAG_02293* qRT-PCR | GGATGTTGCCTTGGCTTAGG |
| UQ3340 | *CNAG_02295* qRT-PCR | CGGAAATAAGCAAGCTGTTTGG |
| UQ3341 | *CNAG_02295* qRT-PCR | AAGGCGGAACAGCATCCA |
| UQ3342 | *ADE2* complementation in genomic location | CAGCTCACATCCTCGCAGCGGGAAAAGCACACATGCCGCC |
| UQ3343 | *ADE2* complementation in genomic location | GGCGGCATGTGTGCTTTTCCCGCTGCGAGGATGTGAGCTG |
| UQ3344 | *ADE2* complementation in genomic location | CCGGGGATGGGAAAGGAAGTGGTTTATCTGTATTAACACG |
| UQ3345 | *ADE2* complementation in genomic location | CGTGTTAATACAGATAAACCACTTCCTTTCCCATCCCCGG |
| UQ3346 | *ADE2* complementation in genomic location | CCCACCATGGCGATGCCAAGG |
| UQ3348 | Multiplex PCR primer | ACTGGTGAGTACTCAACCAAG |
| UQ3504 | *CNAG_05180* qRT-PCR | CCACGCTGCTGTCATGGAT |
| UQ3505 | *CNAG_05180* qRT-PCR | CTGGACACCGCGGTCTTC |
| UQ3506 | *CNAG_05182* qRT-PCR | CTAGACTCCCTCGAGCACATACC |
| UQ3507 | *CNAG_05182* qRT-PCR | AATTGCAAGTCACCATGGTTGTC |
| UQ3508 | *CNAG_01522* qRT-PCR | CGTCTCCCTTATTGACGAATTCA |
| UQ3509 | *CNAG_01522* qRT-PCR | AGTCGGTAGCATCGGGATTG |
| UQ3510 | *CNAG_01524* qRT-PCR | CTGAACAGCTGGAGAAGGACAA |
| UQ3511 | *CNAG_01524* qRT-PCR | TCTCACTCCGTTCCCGAATC |
| UQ3512 | 5' region for subcloing HYG and NEO | CGTTGACGAATGGTGTGATGG |
| UQ3513 | 5' region for subcloing HYG and NEO | CGTGTTAATACAGATAAACCGGCCAATGAAGCACGCTCAA |
| UQ3514 | Marker region for subcloing HYG and NEO | TTGAGCGTGCTTCATTGGCCGGTTTATCTGTATTAACACG |
| UQ3515 | Marker region for subcloing HYG and NEO | GAGATAGGGTTGAGTCCATGGCTGCGAGGATGTGAGCTGG |
| UQ3516 | 3' region for subcloing HYG and NEO | CCAGCTCACATCCTCGCAGCCATGGACTCAACCCTATCTC |
| UQ3517 | 3' region for subcloing HYG and NEO | AGCGCAACGCAATTAATGTGA |
